# Predictions of Liquid Methane (LCH_4_) Lubricated Hybrid Tilting Pad Journal Bearings for Reusable Rocket Turbopumps

**DOI:** 10.3390/ma19112285

**Published:** 2026-05-28

**Authors:** Youngwoo Kim, Tae Ho Kim

**Affiliations:** 1 Department of Mechanical Engineering, Kookmin University, Seoul 02707, Republic of Korea; kywoo123123@kookmin.ac.kr; 2 School of Mechanical Engineering, Kookmin University, Seoul 02707, Republic of Korea

**Keywords:** liquid methane, hybrid tilting pad journal bearing, load capacity, rotordynamic coefficients, turbopump

## Abstract

**Highlights:**

- LCH_4_ property variations strongly affected bearing pressure and temperature.
- Low-viscosity LCH_4_ required hydrostatic support to improve load capacity.
- Supply pressure ratio most strongly influenced LCH_4_ bearing performance.
- Larger recess area weakened hydrostatic support and dynamic coefficients.
- The results support preliminary design of reusable rocket turbopumps in LCH_4_-lubricated condition.

**Abstract:**

This paper presents a performance analysis of a hybrid tilting pad journal bearing (TPJB) for reusable liquid methane (LCH_4_) turbopumps. The numerical model incorporates temperature- and pressure-dependent density and viscosity of LCH_4_ using fourth-order polynomial correlations based on the National Institute of Standards and Technology (NIST) data. A bulk-flow thermohydrodynamic analysis solves the Reynolds and energy equations considering turbulence, compressibility, recess inertia effects, and thermal mixing. The model calculates the pressure and temperature fields using the finite element and finite difference methods, respectively. The results of the static load and length-to-diameter ratio identify the available load range for the present bearing geometry. The cryogenic LCH_4_ supplied through the recess locally suppresses temperature rise and produces spatial variations in its density and viscosity. The preload and radial clearance design can compensate for the limited load-carrying capacity of low-viscosity LCH_4_, while they also can increase friction coefficient and temperature rise. The supply pressure ratio is the dominant parameter because it strengthens hydrostatic support and compensates for the weak hydrodynamic effect of LCH_4_. In contrast, larger recess area weakens hydrostatic support and reduces dynamic coefficients. These results provide bearing-level design guidance for reusable LCH_4_ turbopumps.

## 1. Introduction

Fluid-film bearings are promising components to support cryogenic turbopumps for reusable rocket engines over rolling element bearings which have limited service life. In particular, hydrostatic bearings operating in a full film regime under appropriate conditions can minimize surface contact and thereby substantially reduce wear, thus enhancing durability and long-term reliability [1,2].

Numerous studies have been conducted on fluid lubricated bearings applicable to cryogenic turbopumps. In 1983, Hannum and Nielson [3] demonstrated the feasibility of a hybrid bearing system operating in liquid hydrogen (LH_2_) by combining angular contact ball bearings with hydrostatic fluid-film support. However, this configuration still relied on ball bearings as mechanical support elements. Therefore, the contact wear and life limitations of rolling-element bearings were not fully removed. This limitation motivates the investigation of full fluid-film bearing concepts for long-life cryogenic turbopump applications. Subsequently, San Andrés [4], Yang et al. [5,6], and Franchek et al. [7] analytically investigated the static and dynamic characteristics of hydrostatic bearings under different fluid environments and identified key design guidelines for turbopump applications. However, such hydrostatic bearings may not fully eliminate a bearing instability in high-speed turbopump designs, as cross-coupled stiffness generated by the fluid-film pressure response can still influence the bearing stability. Another remarkable type of bearing is a foil bearing, offering long operational life and providing the required stiffness and damping characteristics to achieve optimal rotordynamic performance of turbopump rotors [8,9]. San Andrés [10] analyzed the isothermal turbulent bulk-flow of liquid oxygen (LOX) foil bearings, finding that structural compliance and hysteretic damping suppress cross-coupled stiffness and prevent half-whirl instability while maintaining adequate load capacity. Behera and Khamari [11] numerically analyzed bump type gas foil bearings for a cryogenic LH_2_ turbopump and predicted sufficient load capacity and stable rotordynamic behavior at 38,000 rpm. Using neural-network modeling and Sobol sensitivity analysis, they identified the eccentricity ratio and number of bearings as key parameters influencing stiffness and damping, confirming the feasibility of foil bearings for high speed cryogenic turbomachinery. However, those foil bearings still have several limitations for cryogenic turbopumps. Their load capacity can remain lower than that of rigid bearings with the same geometry and depends strongly on speed-dependent hydrodynamic film formation. Their stiffness and damping also depend on the foil compliance, bump stiffness, and structural damping, which complicates accurate prediction. These limitations motivate the investigation of a hybrid tilting pad journal bearing (TPJB), which combines externally pressurized hydrostatic support, hydrodynamic pressure generation, and pad self-alignment to improve load capacity and rotordynamic stability under cryogenic turbopump conditions. San Andrés [12] predicted the cryogenic performance of flexure-pivot hybrid TPJB and showed that combining hydrostatic and hydrodynamic effects enhances the load-carrying capacity and rotordynamic stability for high-speed turbopump applications.

The growing adoption of liquid methane (LCH_4_) and LOX propellants, which is driven by the high energy density of methane, higher boiling point than other cryogenic fuels, and high thrust per unit mass, has accelerated the development of turbopumps optimized for efficient and durable operation in next generation reusable rocket engines [13]. However, studies on the performance prediction of hybrid TPJB for turbopump applications are still very limited, and in particular, no research incorporating liquid methane lubrication has yet been conducted.

This study conducted a performance analysis of a hybrid TPJB considering both hydrostatic and hydrodynamic effects under LCH_4_ lubrication. The bearing analysis extended the computational model and method established by Mehdi et al. [14,15] by using the mechanical properties of LCH_4_ depending on temperature and pressure. The analysis results of a TPJB with recesses and orifices for jacking-oil supply with incompressible fluids suitably agreed with the experimental results in reference [16]. Furthermore, an analysis model incorporating turbulence and compressibility effects for a TPJB lubricated with supercritical CO_2_ was verified through comparisons with the experimental data in reference [17]. Existing analytical findings indicate that considering hydrostatic effects contributes to increasing the load-carrying capacity with higher supply pressure, suggesting that hydrostatic support can reduce friction during restart and enhance the overall bearing durability.

Various experimental and analytical studies have been conducted to validate the performance and stability of hydrostatic bearings. In rotordynamic analyses of rotor-bearing systems, accurately predicting bearing rotordynamic coefficients, namely, stiffness and damping, is essential for evaluating the system stability and applicability. This study analyzes a hybrid TPJB operating under LCH_4_ lubrication. The model considers hydrostatic and hydrodynamic effects, turbulence, fluid compressibility, and recess inertia effect. The parametric analysis evaluates how the main design and operating parameters affect the bearing static performance and stiffness and damping coefficients. The results provide bearing-level data for future rotor-bearing analysis of reusable LCH_4_ turbopumps.

## 2. Analytical Model

### 2.1. Hybrid TPJB Lubricated with LCH_4_

**Figure 1:**
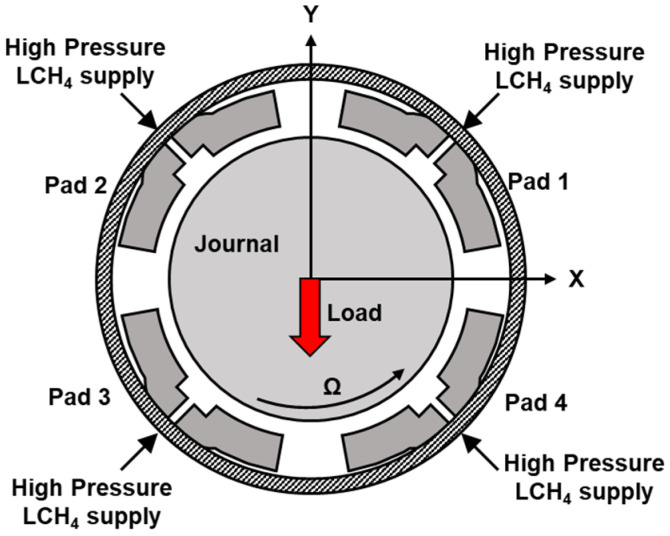
Schematic of hybrid tilting pad journal bearing (TPJB) lubricated with high pressure liquid methane (LCH_4_) in load-between-pad configuration with recesses.

Figure 1 illustrates a schematic of the analyzed hybrid TPJB. High pressure LCH_4_ enters each pad and supports the rotating shaft, and the bearing operates in a load-between-pad (LBP) configuration, where the applied load acts between adjacent pads. A rocker-back pivot supports each pad and allows it to rotate. An orifice and a rectangular recess at the center of each pad deliver pressurized LCH_4_ through the pivot. The supplied fluid develops a pressure field within the clearance between the rotating shaft and the pads and generates a lubricating film that enables smooth operation.

### 2.2. Polynomial Fitting for LCH_4_

**Figure 2:**
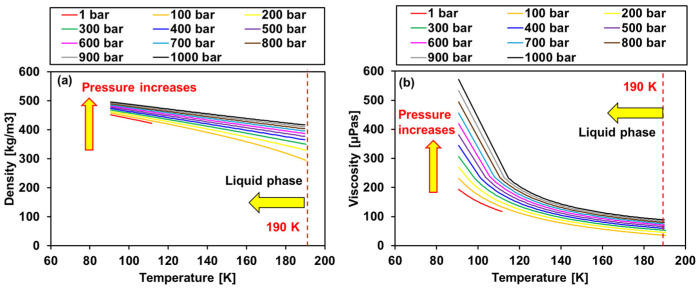
(**a**) Density and (**b**) viscosity of LCH_4_ reported by the National Institute of Standards and Technology (NIST) according to temperature for various pressures.

Recently, reference [18] reported that the turbopump inlet conditions are around 3 bar and 100 K, and the pressure can rise up to approximately 640 bar within the pump stages in typical LCH_4_ turbopumps. Therefore, significant variations in LCH_4_ density and viscosity are expected under such operating conditions. In the hybrid TPJB, the high pressure LCH_4_ supplied through the recess, the hydrodynamic pressure generated within the lubricant film, and the shear-induced temperature rise produce local pressure and temperature variations inside the bearing clearance. Thus, the present model uses the thermophysical properties of the density and viscosity of LCH_4_ reported by the National Institute of Standards and Technology (NIST) to investigate the influence of temperature and pressure, as shown in Figure 2 [19]. Figure 2 shows that LCH_4_ density and viscosity decrease with increasing temperature and increase with rising pressure over the liquid-phase range. For density, the effect of pressure becomes more pronounced at higher temperatures. For viscosity, the pressure-induced increase is more notable at lower temperatures. To quantitatively capture these characteristics, polynomial curve fitting describes the temperature- and pressure-dependent variations in density and viscosity. Equation (1) and Appendix A provide the resulting polynomial fitting equations for both properties:

*f*(*T,P*) = *C*_00_ + *C*_01_*P* + *C*_02_*P*^2^ + *C*_03_*P*^3^ + *C*_04_*P*^4^ + (*C*_10_ + *C*_11_*P* + *C*_12_*P*^2^ + *C*_13_*P*^3^)*T* + (*C*_20_ + *C*_21_*P* + *C*_22_*P*^2^)*T*^2^ + (*C*_30_ + *C*_31_*P*)*T*^3^ + *C*_40_*T*^4^ (1)

where *T* and *P* are the temperature and pressure of the LCH_4_, respectively; see Appendix A for the values of the coefficients (*C_ij_*, *i*,*j* = 0, …, 4).

**Figure 3:**
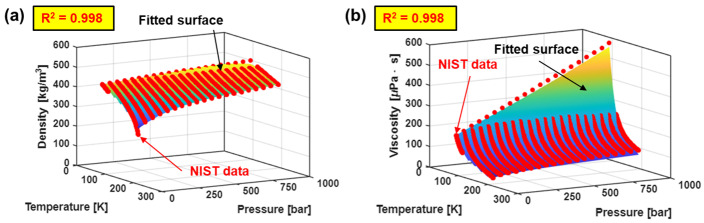
Comparison of NIST data and polynomial fitting model for (**a**) density and (**b**) viscosity using three-dimensional surface plots.

Figure 3 shows the LCH_4_ density and viscosity obtained from the NIST data and those calculated using the polynomial fitting in three-dimensional plots according to temperature and pressure. The coefficients of determination (*R*^2^) for the fitted density and viscosity models are both 0.998. Therefore, the derived polynomial fitting models accurately describe the reference data. Accordingly, the model captures temperature- and pressure-dependent variations of LCH_4_ properties within the bearing clearance. This approach updates the local density and viscosity of LCH_4_ according to the calculated pressure and temperature fields. The updated properties directly affect the pressure distribution, temperature rise, and stiffness and damping coefficients used in the bearing performance evaluation.

## 3. Numerical Method

### 3.1. Thermohydrodynamic Model for Turbulent Compressible Fluid Flows

**Figure 4:**
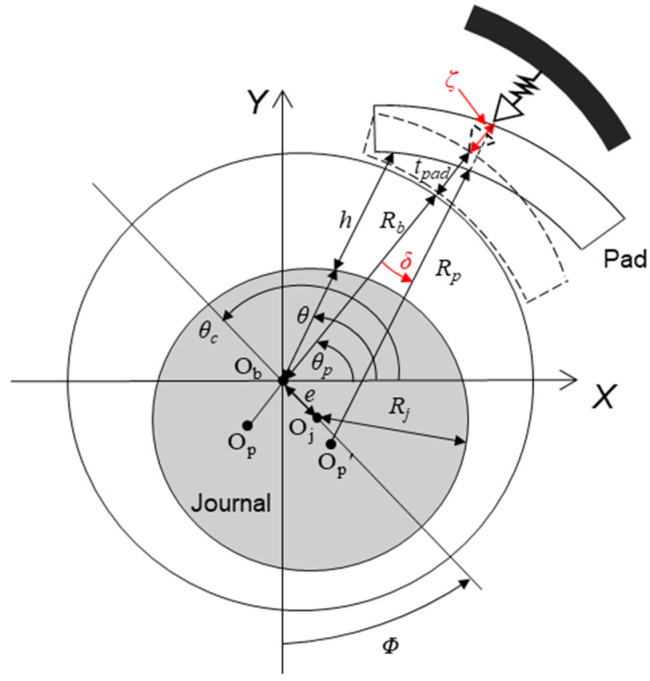
Geometry of TPJB considering pad rotation and pivot deformation.

Figure 4 illustrates the motion of the pad within the TPJB under pivot deformation, where *R_p_*, *R_j_*, *R_b_*, *t_pad_*, *e*, *h*, *θ_c_*, *θ_p_*, and *θ* are the pad curvature radius, journal radius, bearing radius, pad thickness, journal eccentricity, film thickness, journal attitude angle, pad pivot angular position from the circumferential origin, and circumferential coordinate measured from the *X* axis, respectively. In addition, *δ* and *ζ* are the pad tilting angle and radial displacement of the pivot. The hydrodynamic bearing analysis begins with the definition of the lubricant film thickness. Equation (2) describes the modified film thickness [20] involving recess depth *D_r_* not considered in the land region of the film:

*h* = *C_p_* + *e*cos(*θ_c_* − *θ*) − (*r_p_* − *ζ*)cos(*θ* − *θ_p_*) − *δ*(*R_p_* + *t_pad_*)sin(*θ* − *θ_p_*) + *D_r_* (2)

where *C_p_* and *r_p_* are the radial clearance defined by the difference between the radii of the pad curvature and journal, and the preload defined as the difference between the radii of the pad curvature and bearing, respectively. The pivot undergoes radial deformation *ζ* under the applied load, and a Hertzian contact-based stiffness model described in references [21,22,23] evaluates this deformation. This study directly adopts the rocker-back pivot stiffness formulation presented in reference [24].

The Reynolds equation that governs the pressure distribution in the pad land region of the hybrid TPJB incorporates the effects of turbulence and fluid compressibility. LCH_4_ is treated as a compressible fluid because its viscosity and density vary considerably with temperature and pressure. This assumption is particularly important under extreme conditions, such as high pressure, cryogenic temperature, and high rotating speed, which lead to large pressure and temperature gradients within the bearing and non-negligible density variations. Furthermore, LCH_4_ exhibits very low viscosity during operation, resulting in a high Reynolds number within the lubricant film. Hence, flow transitions can occur beyond the laminar regime, and turbulence effects should be included to accurately predict the pressure distribution under high speed operation. Although fluid inertia is also a factor that should be considered under such operating conditions, previous results reported in reference [25] indicate that its influence becomes less significant in the low Sommerfeld number region. Therefore, the present analysis neglects inertia terms in the Reynolds equation for the continuous film region to reduce computational complexity:

(3) $$\frac{\partial }{\partial x}\left(\frac{G_{x}\rho h^{3}}{\mu }\frac{\partial p}{\partial x}\right)+\frac{\partial }{\partial z}\left(\frac{G_{z}\rho h^{3}}{\mu }\frac{\partial p}{\partial z}\right)=\frac{U}{2}\frac{\partial \left(\rho h\right)}{\partial x}+\frac{\partial \left(\rho h\right)}{\partial t}$$

where ρ, *h*, μ*, p*, *U*, and *t* are the lubricant local density, film thickness, local viscosity, pressure, journal surface velocity, and time, respectively, *x* and *z* represent the circumferential and axial directions, respectively [26]. *G_x_* and *G_z_* are the turbulence coefficients calculated using the Hirs bulk-flow model [27]. The local Reynolds number, Re = ρUh/μ, is determined from local density ρ, viscosity μ and film thickness h within the lubricant film:

(4) $$G_{x}\;=\;\frac{2^{1+m_{0}}}{n_{0}(2\;+\;m_{0})}Re^{-(1+m_{0})},\;G_{z}\;=\;\frac{2^{1+m_{0}}}{n_{0}}Re^{-(1+m_{0})}$$

where the empirical constants $m_{0}$ and $n_{0}$ take values of −0.25 and 0.066, respectively. Under laminar flow, where the Reynolds number is below the critical value of 1000 for turbulence onset, both $G_{x}$ and $G_{z}$ reach their maximum of 1/12. Consequently, the governing equations converge to the classical Reynolds equation valid under laminar flow.

The energy transport equation based on the bulk-flow theory allows for the calculation of the temperature distribution within the bearing. The first term on the left hand side represents the convective transport of energy by fluid flow, while the second and third terms correspond to convective heat transfer to the bearing and journal surfaces, respectively. These terms balance on the right hand side by the compressive work and viscous energy dissipation resulting from shear friction within the lubricant film [28]:

(5) $$c_{p}\left[\frac{\partial \left(\rho \mathrm{hVT}\right)}{\partial x}\;+\;\frac{\partial \left(\rho \mathrm{hWT}\right)}{\partial z}\right]+h_{b}\left(T\;-{\;T}_{b}\right)+h_{j}\left(T\;-{\;T}_{j}\right)\;={\;\beta }_{t}\mathrm{hT}\left[V\frac{\partial p}{\partial x}\;+\;W\frac{\partial p}{\partial z}\right]\;+\;\frac{\mathrm{hU}}{2}\frac{\partial p}{\partial x}\;+\;\frac{\mu }{h}\left[\frac{1}{G_{x}}\left(V^{2}\;+\;W^{2}\;+\;\frac{U}{2}V\right)\;+\;\frac{U}{G_{x}}\left(\frac{U}{4}\;-\;V\right)\right]$$

where *V*, *W*, *h_b_*, and *h_j_* are the circumferential and axial bulk-flow velocities across the lubricant film, the convective heat transfer coefficients at the bearing and journal surfaces with surface temperatures *T_b_* and *T_j_*, respectively, and *β_t_* is the volumetric expansion coefficient equal to 1 for an ideal gas and 0 for an incompressible fluid.

**Figure 5:**
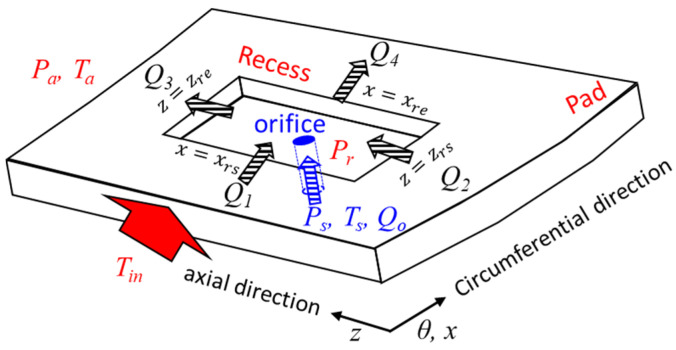
Schematic of a single pad with the recess pocket and orifice restrictor.

In the TPJB, high pressure LCH_4_ enters the recess region of each pad through an orifice restrictor, as shown in Figure 5. Each pad contains a recess that extends from $x_{\mathrm{rs}}$ to $x_{\mathrm{re}}$ in the circumferential direction and from $z_{\mathrm{rs}}$ to $z_{\mathrm{re}}$ in the axial direction. The supply pressure ($P_{s}$) drops to the recess pressure ($P_{r}$) within the recess before flowing through the land region. The recess pressure is determined using the flow balance condition between the inflow through the orifice ($Q_{o}$) and the net outflow across the recess boundary ($Q_{r}$), as expressed in Equation (6):

(6) $$Q_{o}-Q_{r}=0$$

(7) $$Q_{o}=C_{d}A_{0}\sqrt{\frac{2}{\rho }\left(P_{s}-P_{r}\right)}$$

(8) $$Q_{r}=\int_{z_{\mathrm{rs}}}^{z_{\mathrm{re}}}\left({\left[-\frac{{G_{x}\rho h}^{3}}{\mu }\frac{\partial p}{\partial x}+\frac{\rho \mathrm{Uh}}{2}\right]}_{x_{\mathrm{re}}}\;-\;{\left[-\frac{G_{x}{\rho h}^{3}}{\mu }\frac{\partial p}{\partial x}+\frac{\rho \mathrm{Uh}}{2}\right]}_{x_{\mathrm{rs}}}\right)\mathrm{dz}\;+\int_{x_{\mathrm{rs}}}^{x_{\mathrm{re}}}\left({\left[-\frac{{G_{z}\rho h}^{3}}{\mu }\frac{\partial p}{\partial z}\right]}_{z_{\mathrm{re}}}\;-\;{\left[-\frac{{G_{z}\rho h}^{3}}{\mu }\frac{\partial p}{\partial z}\right]}_{z_{\mathrm{rs}}}\right)\mathrm{dx}$$

where *C_d_* and *A*_0_ in Equation (7) are the discharge coefficient and orifice area, respectively. Equation (8) determines the net outflow from the recess by integrating the velocity components along the circumferential and axial recess boundaries; that is, $Q_{r}\;=\;Q_{4}-Q_{1}\;+\;Q_{3}-Q_{2}$.

In addition, the model accounts for inertial effects at the circumferential and axial recess boundaries by applying a Bernoulli-based model [29], which expresses the pressure drop across the recess boundaries to the mean fluid velocity as follows:

(9) $$\{\begin{array}{c} P_{r}\;-\;P_{\mathrm{dc}}\;=\;\frac{\rho_{l}}{2}Q_{l}^{2}\left(\frac{1}{h_{l}^{2}}\;-\;\frac{1}{h_{r}^{2}}\right)\;+\;0.803\rho_{l}Q_{l}^{2}\left(\frac{1}{Re_{l}^{0.43}h_{l}^{2}}\;-\;\frac{1}{Re_{r}^{0.43}h_{r}^{2}}\right)\;-\;1.53\rho_{l}V^{2}\left(\frac{1}{Re_{l}^{0.367}}\;-\;\frac{1}{Re_{r}^{0.367}}\right)\\ P_{r}\;-\;P_{\mathrm{da}}=\frac{\rho_{l}}{2}Q_{l}^{2}\left(\frac{1}{h_{l}^{2}}\;-\;\frac{1}{h_{r}^{2}}\right) \end{array}$$

where $P_{\mathrm{dc}}$, $P_{\mathrm{da}}$, and $Q_{l}$ are the pressure at the entrances of the circumferential film land, pressure at the entrances of the axial film land, and flux at the land area, respectively, and subscripts l and r indicate the land and recess sides at the recess boundary, respectively.

The boundary conditions required for the numerical calculation of pressure and temperature distributions are as follows:

(10) $$\{\begin{array}{c} P\left(x_{s},\;z\right)\;=\;P\left(x_{e},z\right)\;=\;P\left(x,\frac{L}{2}\right)\;=\;P\left(x,-\frac{L}{2}\right)\;=\;P_{a}\\ P\left(x,z\right)\;=\;P_{r}\;\left(x_{\mathrm{rs}}\;<\;x\;<\;x_{\mathrm{re}},\;z_{\mathrm{rs}}\;<\;z\;<\;z_{\mathrm{re}}\right)\\ T_{b}\;=\;T_{j}\;=\;T_{s} \end{array}$$

where $x_{s}$ and $x_{e}$ denote the start and end positions of the pad and follow from $R_{j}\theta_{s}$ and $R_{j}\theta_{e}$, respectively. $\theta_{s}$, $\theta_{e}$, L, $P_{a}$, and $T_{s}$ are the pad start and end angles, pad axial length, ambient pressure, and supply temperature, respectively. The pressures at all pad boundaries are equal to the ambient pressure, and the pressure within the recess region sets the recess pressure. The surface temperatures of both the bearing and journal assume the lubricant supply temperature. The inlet temperature ($T_{\mathrm{in}}$) for the leading edge of each pad is calculated by using the thermal energy mixing condition [30], which considers mixing of hot lubricant discharged from the upstream region with the newly supplied cold lubricant.

The static performance and dynamic coefficients of the bearing are obtained by integrating the pressure field over each pad surface. The load-carrying capacity of the bearing equals the sum of the static pressure integrals over the film region between the start and end points of each pad, as expressed in Equations (11) and (12):

(11) $$\sum F_{X}\;=\;\sum_{i=1}^{N_{\mathrm{pads}}}{\left[\int_{-\frac{L}{2}}^{\frac{L}{2}}\int_{x_{s}}^{x_{e}}\left(P\cos\theta \right)\mathrm{dx}\mathrm{dz}\right]}_{i}\;=\;W_{X}$$

(12) $$\sum F_{Y}\;=\;\sum_{i=1}^{N_{\mathrm{pads}}}{\left[\int_{-\frac{L}{2}}^{\frac{L}{2}}\int_{x_{s}}^{x_{e}}\left(P\sin\theta \right)\mathrm{dx}\mathrm{dz}\right]}_{i}\;-\;W_{b}=W_{Y}\;-\;W_{b}$$

where *W_X_*, *W_Y_*, and *W_b_* are the horizontal and vertical reaction forces, and applied static load. The hydrodynamic pressure distributed over the lubricant film of each pad generates moments for each pivot, causing the pads to tilt until reaching moment equilibrium. Moment equilibrium for the *i*th pad is expressed as follows:

(13) $$M_{i}\;=\;\int_{-\frac{L}{2}}^{\frac{L}{2}}\int_{x_{s}}^{x_{e}}(R_{j}(\theta \;-\;\theta_{p})\left(P\cos\left(\theta \;-\;\theta_{p}\right)\right)\mathrm{dx}\mathrm{dz}$$

where *M_i_* represents the moment acting on the *i*th pad about its pivot. The frictional losses generated in turbopump bearings are not merely energy dissipation factors but critical design considerations that directly influence the lubrication conditions, heat generation characteristics, component lifespan, and overall system efficiency. Therefore, analyzing friction losses across the entire operating speed range is essential to quantitatively evaluate loss characteristics and guide the optimization of cooling design, lubricant supply conditions, and pad geometry. This analysis allows for enhancing the bearing performance and contributes to improving the long-term reliability of a turbopump and overall efficiency of a launch vehicle propulsion system. The friction coefficient, f, is expressed as follows:

(14) $$f\;=\;\frac{1}{\omega R_{j}W_{b}}\sum_{i=1}^{N_{\mathrm{pads}}}\int_{-\frac{L}{2}}^{\frac{L}{2}}\int_{x_{s}}^{x_{e}}\left(\frac{\mathrm{hU}}{2}\frac{\partial p}{\partial x}\;+\;\frac{\mu }{h}\left[\frac{1}{G_{x}}\left(V^{2}\;+\;W^{2}\;+\;\frac{U}{2}V\right)\;+\;\frac{U}{G_{x}}\left(\frac{U}{4}\;-\;V\right)\right]\right)\mathrm{dx}\mathrm{dz}$$

where *ω* denotes the angular velocity of the journal. The integral term represents the power loss due to viscous shear within the lubricant film, and this power loss determines the friction coefficient.

The stiffness and damping coefficients of the bearing are expressed as Equation (15) from the ratio of changes in bearing reaction forces in the horizontal (*X*) and vertical (*Y*) directions to small perturbations in displacement and velocity about the journal center at static equilibrium.

(15) $$K_{\mathrm{XX}}=\frac{\Delta W_{X}}{\Delta X},\;K_{\mathrm{XY}}=\frac{\Delta W_{X}}{\Delta Y},\;K_{\mathrm{YX}}=\frac{\Delta W_{Y}}{\Delta X},\;K_{\mathrm{YY}}=\frac{\Delta W_{Y}}{\Delta Y},\;C_{\mathrm{XX}}=\frac{\Delta W_{X}}{\Delta \dot{X}},\;C_{\mathrm{XY}}=\frac{\Delta W_{X}}{\Delta \dot{Y}},\;C_{\mathrm{YX}}=\frac{\Delta W_{Y}}{\Delta \dot{X}},\;C_{\mathrm{YY}}=\frac{\Delta W_{Y}}{\Delta \dot{Y}}$$

### 3.2. Hybrid TPJB Analysis

**Figure 6:**
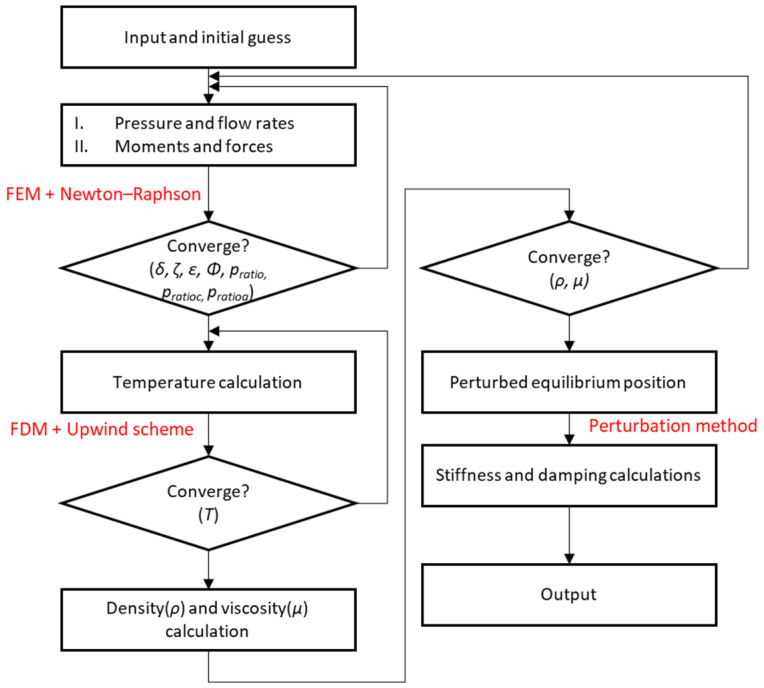
Flowchart of numerical procedure for hybrid TPJB analysis.

Figure 6 shows a flowchart of the overall numerical procedure for determining the static equilibrium position of the TPJB, followed by the calculation of temperature and pressure distributions and evaluation of bearing stiffness and damping coefficients. The analysis begins with the bearing geometry, initial lubricant properties and initial estimates of the pad tilting angle, pivot deformation, journal eccentricity ratio, journal attitude angle, and pressure ratios $p_{\mathrm{ratio}}$ ($P_{r}/P_{s}$), $p_{\mathrm{ratioc}}$ ($P_{\mathrm{dc}}/P_{s}$), and $p_{\mathrm{ratioa}}$ ($P_{\mathrm{da}}/P_{s}$). The hydrodynamic pressure acting on each pad is obtained by numerically solving Equation (3) using a finite element method. The resulting fluid-induced load generates a restoring force at the pivot, and the solution must satisfy the following static equilibrium conditions:

(16) $$\left(\begin{array}{c} \begin{array}{c} {\left(F_{\mathrm{pad}}\;-\;F_{p}\right)}_{n=1\ldots N_{\mathrm{pad}}}\\ M_{n=1\ldots N_{\mathrm{pad}}} \end{array}\\ \sum F_{X}\\ \sum F_{Y}\\ {\left(Q_{o}\;-\;Q_{r}\right)}_{n=1\ldots N_{\mathrm{pad}}}\\ {\left(P_{dc_{i}}\;-\;P_{dc_{i-1}}\right)}_{n=1\ldots \mathrm{Npad}}\\ {\left(P_{da_{i}}\;-\;P_{da_{i-1}}\right)}_{n=1\ldots \mathrm{Npad}} \end{array}\right)=\left(\begin{array}{c} \begin{array}{c} 0\\ 0 \end{array}\\ 0\\ 0\\ 0\\ 0\\ 0 \end{array}\right)$$

The fluid-film load on each pad ($F_{\mathrm{pad}}$) must be balanced with the corresponding pivot restoring force ($F_{p}$), and each pad moment and sum of horizontal bearing forces must be zero. In addition, the vertical bearing reaction force must be in equilibrium with the applied static load. The flow rate supplied through the orifice must also equal the total outflow along the recess boundary. The circumferential and axial pressure drops at the recess boundaries iteratively update until convergence. The subscript i denotes the current iteration step and i − 1 represents the previous step in the recess pressure update. During this iterative Newton–Raphson procedure, the calculation proceeds until the recess pressure converges to a value that satisfies both the flow rate equilibrium condition and the circumferential and axial pressure drops across the recess boundaries. To solve these coupled equations, the Newton–Raphson numerical method determines the static equilibrium position. Subsequently, this model calculates the lubricant inlet temperature at the pad leading edge using a thermal energy mixing formulation and computes the two dimensional temperature field by solving the energy transport equation with a finite difference upwind scheme. The temperature field, together with the pressure distribution, update the lubricant density and viscosity iteratively until convergence. Finally, at the converged static condition, a small perturbation to the journal position computes a new equilibrium state, and the resulting differences in reaction forces between the two positions determine the stiffness and damping coefficients of the bearing.

### 3.3. Bearing, Pivot, and Recess Geometries, and Operating Condition

Table 1 lists the geometric parameters of the considered TPJB. The bearing consists of four pads and employs the LBP configuration. Both the bearing diameter and axial pad length are 50 mm. Each pad has an angular extent of 70°, and both the pivot offset and preload are set to 0.5. The pad thickness and radial clearance are 16 mm and 75 μm, respectively. The rocker-back pivot has housing and pivot radii of 42 and 36 mm, respectively, and the pivot length is equal to the bearing length. Each of the four pads contains a rectangular recess at its geometric center, with an orifice at the recess center. The circumferential and axial lengths of the recess are 10 mm, and the orifice diameter is 1 mm. The recess depth and orifice discharge coefficient are set to 0.2 mm and 0.68, respectively.

The corresponding lubricant properties, operating conditions and analysis parameters are listed in Table 2. Considering the extreme cryogenic and high pressure operation environment of turbopump bearings, the ambient pressure and temperature are set to 200 bar and 112 K, respectively, obtaining density and viscosity of LCH_4_ of 438.4 kg/m^3^ and 150 μPa·s, respectively. The supply pressure of LCH_4_ delivered through the orifice is set to 400 bar. In addition, the parameter ranges influencing the bearing performance, such as static load, rotating speed, length-to-diameter ratio, preload, radial bearing clearance ratio, supply pressure ratio, and ratio of recess area to total bearing area are set for the analysis. At rotational speeds of 30,000 and 60,000 rpm, the nominal Reynolds numbers based on the initial conditions are 8608 and 17,216, respectively. Therefore, the flow conditions indicate that turbulence modeling is necessary to accurately capture the fluid behavior under high speed cryogenic operating conditions. A small differential step of 10^−6^ is set to compute the Jacobian numerically, while the perturbation step ratio is set to 0.001. The perturbation displacement equals the product of the perturbation step ratio and the bearing radial clearance, and the perturbation velocity equals the product of the displacement and the angular velocity. The mesh consists of 12 circumferential and 20 axial elements, and the static and dynamic convergence tolerances for all iterative calculations are set to 10^−6^ and 10^−8^, respectively.

The present study defines the dimensionless parameters as follows. The Sommerfeld number, which characterizes the operating condition of the bearing, is expressed as Equation (17). Also, the main dimensionless analysis parameters are defined as Equation (18) where ε is the journal eccentricity ratio, $P^{*}$ is the dimensionless pressure normalized by the ambient pressure, and $\Delta T^{*}$ represents the dimensionless temperature rise relative to the reference temperature $T_{a}$. Equation (19) then introduces the corresponding dimensionless stiffness and damping coefficients, obtained by non-dimensionalizing the stiffness and damping terms defined in Equation (15).

(17) $$S=\frac{\mu_{0}\omega \mathrm{DL}}{W_{b}}{\left(\frac{R_{j}}{C_{p}}\right)}^{2}$$

(18) $$\varepsilon =\frac{e}{C_{b}},\;P^{*}=\frac{P}{P_{a}},\;\Delta T^{*}=\frac{T-\;T_{a}}{T_{a}}$$

(19) $$k_{\alpha \beta }=K_{\alpha \beta }\frac{C_{p}}{W_{b}},\;c_{\alpha \beta }=C_{\alpha \beta }\frac{C_{p}\omega }{W_{b}},\;\alpha ,\beta =X,Y$$

## 4. Results

### 4.1. Static Load

**Figure 7:**
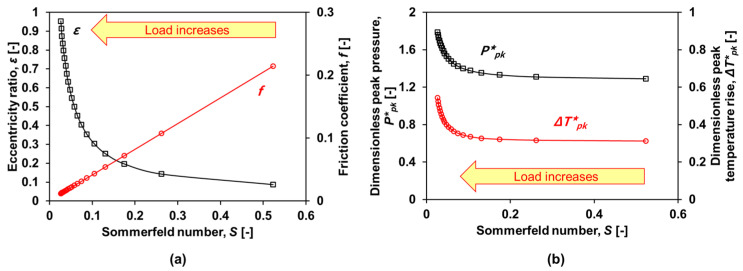
Predicted (**a**) journal eccentricity ratio and friction coefficient and (**b**) dimensionless peak pressure and temperature rise according to the Sommerfeld number at constant rotating speed of 60,000 rpm.

The static load analysis considers the operating conditions of N = 60,000 rpm, L/D = 1, m = 0.5, ψ = 0.0015, $P^{\prime }\;=\;2$, and χ = 0.06. Increasing the applied load from 500 to 10,000 N reduces the corresponding Sommerfeld number from 0.52 to 0.026. As shown in Figure 7a, the journal eccentricity ratio increases and the friction coefficient decreases with increasing static load. The journal attitude angle remains constant at 0° and therefore is not displayed in the figure. At S = 0.026, the journal eccentricity ratio reaches 0.958, approaching the operational limit of the bearing under the given geometry. Beyond this point, the minimum film thickness becomes critically small, posing a risk of shaft–pad contact and potential bearing failure. This behavior is important for LCH_4_-lubricated bearings because the low viscosity of LCH_4_ provides a relatively small hydrodynamic film margin under heavy load operation, making sufficient hydrostatic support essential for maintaining full-film lubrication. Figure 7b shows that the dimensionless peak pressure on the pad increases with static load because the bearing must generate a stronger supporting pressure field in the loaded region. The reduced film thickness also increases the local velocity gradient and intensifies viscous shear within the LCH_4_ film, resulting in higher power loss and a larger dimensionless peak temperature rise. Although the friction coefficient decreases with increasing static load, this trend does not indicate a reduction in shear loss. Instead, the applied load increases more strongly than the viscous power loss in the normalized friction coefficient, producing the decreasing trend. 

**Figure 8:**
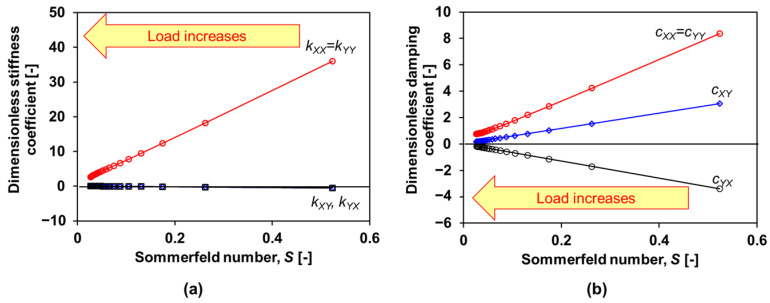
Predicted dimensionless (**a**) stiffness and (**b**) damping coefficients according to the Sommerfeld number at rotating speed of 60,000 rpm.

Figure 8 shows the dimensionless stiffness and damping coefficients according to the Sommerfeld number. The dimensionless direct stiffness components are consistently larger than the cross-coupled stiffness components over the entire load range. This behavior originates from the self-aligning motion of the tilting pads and the hydrostatic support generated in the recesses. Under journal perturbation, the pads adjust the converging film geometry around their pivots, causing the pressure response to act mainly in the displacement direction. The recess pressure further strengthens the direct restoring force, while the pad tilting motion suppresses circumferential pressure asymmetry caused by cross-coupled stiffness [25]. As the Sommerfeld number increases—that is, as the applied load decreases—the separation between the direct and cross-coupled components becomes more pronounced, reflecting the stronger dominance of the direct stiffness and damping terms under light load operating conditions. In other words, the static load primarily affects the direct components rather than the cross-coupled components.

**Figure 9:**
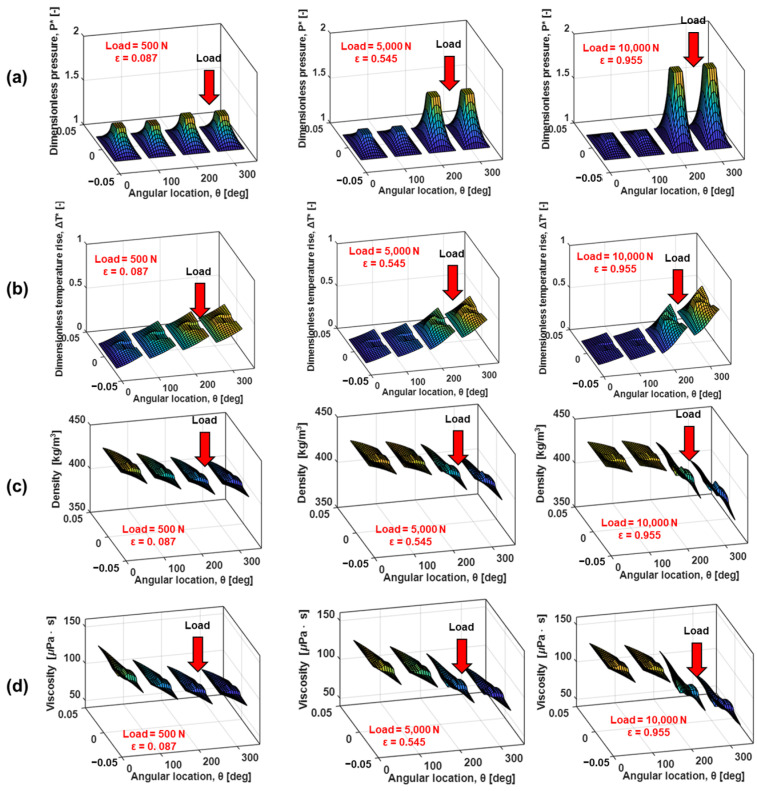
Three-dimensional plots of (**a**) dimensionless pressure, (**b**) dimensionless temperature rise, (**c**) density, and (**d**) viscosity for low, moderate, and high journal eccentricity ratios and three static loads at rotating speed of 60,000 rpm. The contour colors transition from blue to yellow with increasing values.

Figure 9 presents the distributions of dimensionless pressure, dimensionless temperature rise, density, and viscosity across the pad surfaces of the hybrid TPJB at three representative journal eccentricity ratios corresponding to low, moderate, and high static load conditions. As shown in Figure 9a, each pad exhibits a localized pressure peak within the recess region, arising from the hydrostatic pressurization through the orifice restrictor. At near-zero journal eccentricity under minimal load, the recess pressure is distributed nearly uniformly across all four pads, reflecting the concentric journal position. As the static load increases, the pressure peaks on the loaded pads (pads 3 and 4) intensify substantially, while those on the unloaded pads (pads 1 and 2) diminish correspondingly. This redistribution indicates that the hydrostatic support shifts toward the loaded region as the journal displacement increases. 

The temperature rise on each pad is the lowest at the leading edge and the highest at the trailing edge, as shown in Figure 9b. The recess region shows a locally lower temperature rise because the relatively cold LCH_4_ is continuously supplied through the recess. As the load increases and the film thickness diminishes, viscous shear intensifies on the loaded pads, elevating the local temperature rise and steepening the thermal gradient across the pad surface. This thermal response directly affects LCH_4_ properties because both density and viscosity vary with temperature and pressure.

The density and viscosity distributions shown in Figure 9c,d reflect the thermophysical response of LCH_4_ to the evolving pressure and temperature fields. Although pressure also affects the LCH_4_ properties, the temperature rise dominates the local property variation under the present conditions. The recess region shows locally higher density and viscosity because the temperature rise remains relatively low in this region. As the static load increases, the interaction among pressure buildup, shear heating, and LCH_4_ property variation becomes more pronounced. This result confirms the need to couple the pressure field, temperature field, and temperature–pressure-dependent LCH_4_ properties when predicting the performance of LCH_4_-lubricated bearings.

### 4.2. Length-to-Diameter Ratio

**Figure 10:**
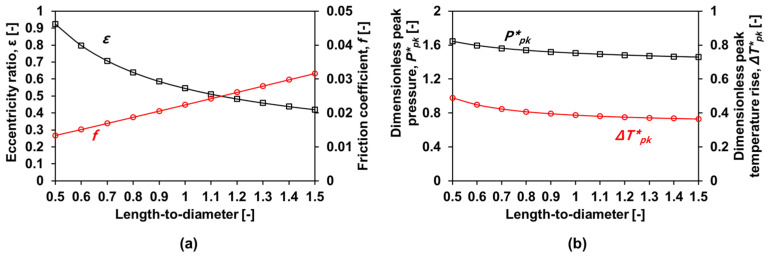
Predicted (**a**) journal eccentricity ratio and friction coefficient and (**b**) dimensionless peak pressure and temperature rise according to length-to-diameter ratio at constant rotating speed of 60,000 rpm and static load of 5000 N.

The analysis of the length-to-diameter ratio considers the operating conditions of $W_{b}\;=\;5000\;N$, N = 60,000 rpm, m = 0.5, ψ = 0.0015, $P^{\prime }\;=\;2$, and χ = 0.06. The study fixes the diameter at 50 mm and varies the bearing length to obtain *L*/*D* ranging from 0.5 to 1.5. As shown in Figure 10a, the journal eccentricity ratio decreases and the friction coefficient increases with increasing *L*/*D*. A larger *L*/*D* increases the effective load-supporting area, thereby improving the load-carrying capacity. This effect is beneficial for LCH_4_ lubrication because the low viscosity of LCH_4_ limits hydrodynamic pressure generation, and the enlarged load-supporting area helps compensate for this limitation. Consequently, as illustrated in Figure 10b, both the dimensionless peak pressure and temperature rise decrease with increasing *L*/*D* because the applied load and heat generation spread over a larger pad surface. Despite the reduction in temperature rise, the friction coefficient increases with increasing *L*/*D*. This behavior occurs because a larger bearing length expands the shearing area between the journal and pads, increasing the total viscous power loss in the LCH_4_ film. At the same time, the increased axial length enhances axial fluid transport and heat spreading, which suppresses local temperature rise and limits temperature-induced variations in LCH_4_ density and viscosity.

**Figure 11:**
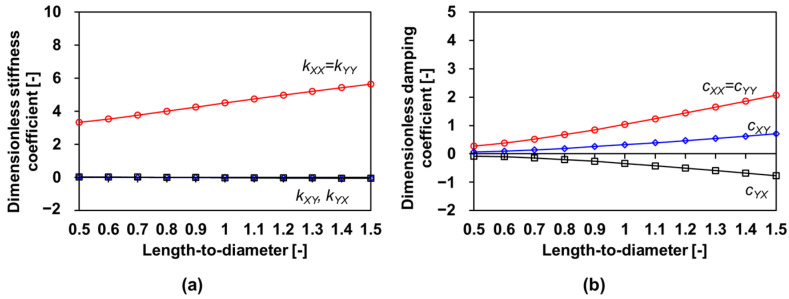
Predicted dimensionless (**a**) stiffness and (**b**) damping coefficients according to length-to-diameter ratio at constant rotating speed of 60,000 rpm and static load of 5000 N.

Figure 11 shows the dimensionless stiffness and damping coefficients according to *L*/*D*. As *L*/*D* increases, the axial pressure distribution becomes more uniform, and the larger reaction area strengthens the fluid-film force response. While the journal eccentricity ratio decreases, as shown in Figure 10, both the dimensionless direct stiffness and damping coefficients tend to increase. As for the damping, the difference between the direct and cross-coupled damping coefficients increases as *L*/*D* increases, indicating that the direct damping component becomes progressively more dominant at higher *L*/*D* values. These results indicate that *L*/*D* has a moderate influence on LCH_4_-lubricated hybrid TPJB performance: it improves load distribution and dynamic coefficients, but its benefit accompanies increased frictional loss due to the larger shearing area.

### 4.3. Preload

**Figure 12:**
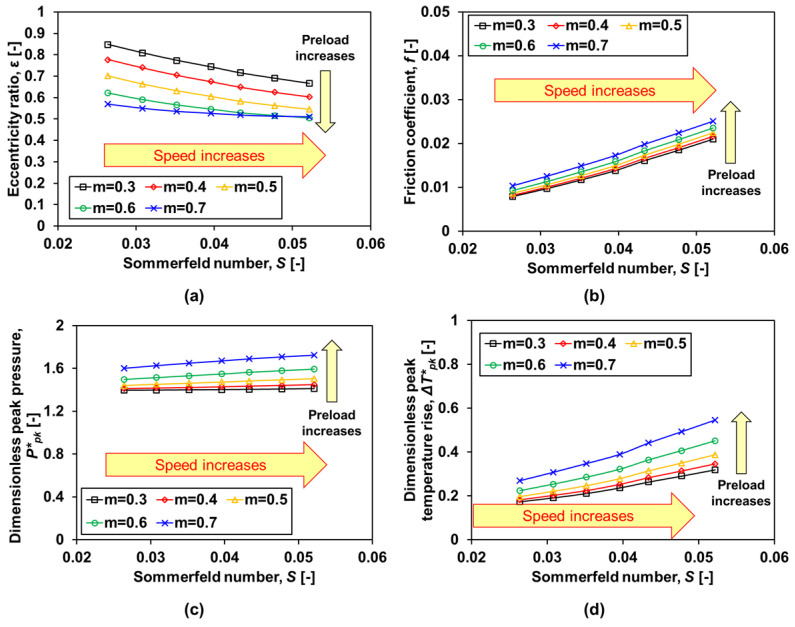
Predicted (**a**) journal eccentricity ratio, (**b**) friction coefficient, (**c**) dimensionless peak pressure, and (**d**) dimensionless peak temperature rise according to the Sommerfeld number under different preload factors for static load of 5000 N.

The preload analysis considers the operating conditions of $W_{b}\;=\;5000\;N$, L/D = 1, ψ = 0.0015, $P^{\prime }\;=\;2$, and χ = 0.06, while varying the preload from 0.3 to 0.7. Increasing the rotational speed from 30,000 rpm to 60,000 rpm raises the corresponding Sommerfeld number from 0.026 to 0.052. The results for journal eccentricity ratio, friction coefficient, dimensionless peak pressure, and dimensionless peak temperature rise according to the Sommerfeld number are shown in Figure 12. For all preload factors, increasing the rotating speed decreases the journal eccentricity ratio, while it increases the friction coefficient, dimensionless peak pressure, and peak temperature rise. This trend indicates that higher journal surface speed strengthens hydrodynamic pressure generation in the low-viscosity LCH_4_ film. As the preload increases, the reduced bearing clearance forms a more converging film geometry and lowers the journal eccentricity ratio over the entire speed range, thereby improving the load-carrying capacity. This effect helps compensate for the limited hydrodynamic pressure generation caused by the low viscosity of LCH_4_. At the same time, the reduced film thickness increases the local velocity gradient and intensifies viscous shear within the LCH_4_ film, leading to increases in both the dimensionless peak pressure and temperature rise. The friction coefficient increases with rotating speed and preload under the fixed bearing geometry. This increase in friction coefficient reflects the enhanced viscous shear and corresponding power loss in the LCH_4_ film, which directly contributes to the observed increase in the dimensionless peak temperature rise.

**Figure 13:**
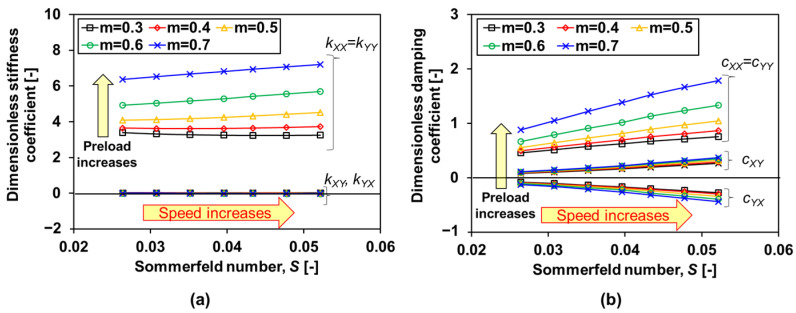
Predicted dimensionless (**a**) stiffness and (**b**) damping coefficients according to the Sommerfeld number under different preload factors for static load of 5000 N.

Figure 13 illustrates the effect of preload on the bearing stiffness and damping. Increasing the preload increases both the dimensionless direct stiffness and damping coefficients because the reduced clearance makes the pressure field more sensitive to small journal displacement and velocity perturbations. This effect is important for LCH_4_-lubricated bearings because the low viscosity of LCH_4_ tends to weaken hydrodynamic pressure generation, whereas higher preload compensates for this limitation by forming a more converging film geometry. Even as the preload increases, the cross-coupled components remain very small because the tilting pads can self-align under journal perturbation. In contrast, the strengthened converging film geometry increases the direct stiffness and damping components. Therefore, the separation between the direct and cross-coupled components becomes larger with increasing preload. This trend supports the use of preload as an effective design parameter for improving rotor-bearing stability.

### 4.4. Radial Bearing Clearance Ratio

**Figure 14:**
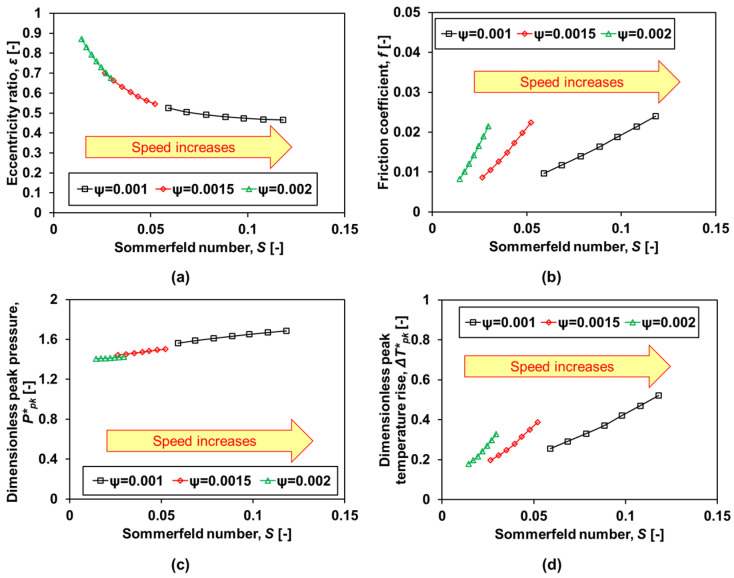
Predicted (**a**) journal eccentricity ratio, (**b**) friction coefficient, (**c**) dimensionless peak pressure, and (**d**) dimensionless peak temperature rise according to the Sommerfeld number under different radial bearing clearance ratio for static load of 5000 N.

The analysis of radial bearing clearance ratio considers the operating conditions of $W_{b}\;=\;5000\;N$, L/D = 1, $P^{\prime }\;=\;2$, and χ = 0.06, while applying three radial clearance ratios of 0.001, 0.0015, and 0.002. These clearance ratios correspond to Sommerfeld number ranges of 0.059–0.12, 0.026–0.052, and 0.014–0.029, respectively. As shown in Figure 14, the results present the journal eccentricity ratio, friction coefficient, dimensionless peak pressure, and dimensionless peak temperature rise as functions of the Sommerfeld number. With increasing rotating speed, the journal eccentricity ratio decreases, whereas the friction coefficient, dimensionless peak pressure, and temperature rise increase. This trend indicates that higher journal surface speed strengthens hydrodynamic pressure generation and shear heating in the low-viscosity LCH_4_ film. As the radial bearing clearance ratio increases, the journal eccentricity ratio increases, while the dimensionless peak pressure and temperature rise decrease. This behavior arises because a larger clearance weakens both hydrostatic and hydrodynamic pressure generation, reducing the load-supporting capability and the associated viscous shear within the lubricant film. In contrast, the friction coefficient remains nearly unchanged over the investigated clearance range. This weak sensitivity indicates that clearance variation does not significantly change the total viscous power loss normalized by the bearing load and journal surface speed, even though it reduces the local shear rate in the LCH_4_ film.

**Figure 15:**
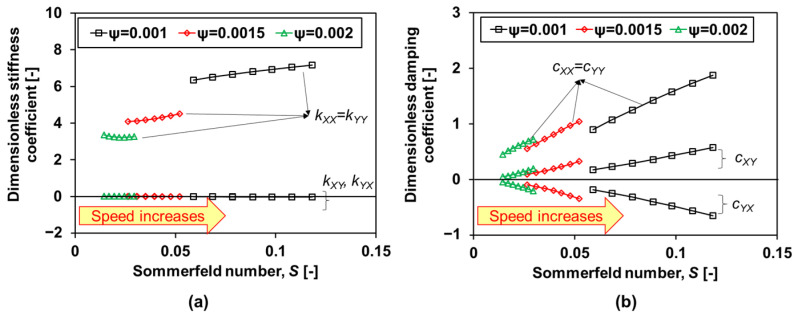
Predicted dimensionless (**a**) stiffness and (**b**) damping coefficients according to the Sommerfeld number under different radial bearing clearance ratio for static load of 5000 N.

Figure 15 further indicates that decreasing the radial clearance ratio increases both the dimensionless direct stiffness and the dimensionless direct damping coefficients. This effect is particularly important for LCH_4_-lubricated bearings because the low viscosity of LCH_4_ makes the dynamic force response more sensitive to clearance selection. Conversely, increasing the radial clearance ratio enlarges the bearing clearance, which requires a higher journal eccentricity to support the same load and weakens both hydrostatic and hydrodynamic pressure contributions. Therefore, radial clearance ratio is one of the most influential design parameters in LCH_4_-lubricated hybrid TPJBs because it strongly affects the balance among load capacity, thermal behavior, and rotordynamic coefficients.

### 4.5. Supply Pressure Ratio

**Figure 16:**
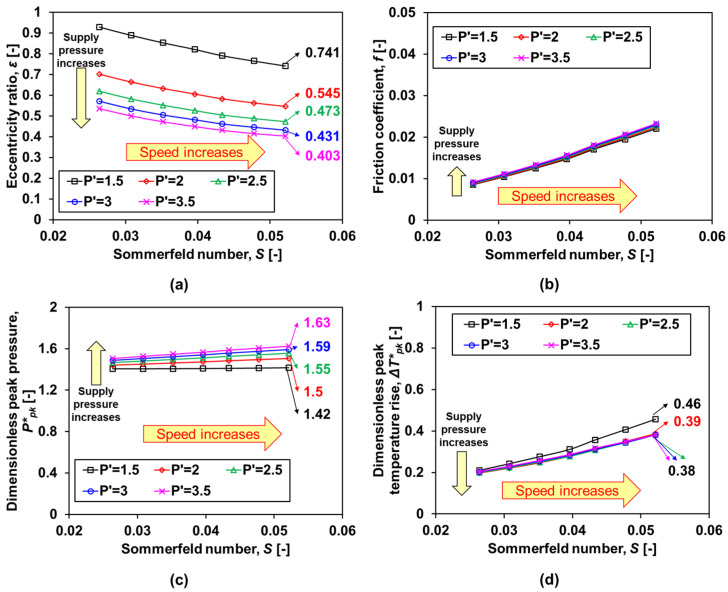
Predicted (**a**) journal eccentricity ratio, (**b**) friction coefficient, (**c**) dimensionless peak pressure, and (**d**) dimensionless peak temperature rise according to the Sommerfeld number under different supply pressure ratios for static load of 5000 N.

The analysis of supply pressure considers the operating conditions of $W_{b}\;=\;5000\;N$, L/D = 1, and m = 0.5, *ψ* = 0.0015, and *χ* = 0.06. The supply pressure ratio is increased from 1.5 to 3.5 at S = 0.052. The resulting journal eccentricity ratio, friction coefficient, dimensionless peak pressure, and dimensionless peak temperature rise according to the Sommerfeld number are shown in Figure 16. As the LCH_4_ supply pressure ratio increases, the journal eccentricity ratio decreases, while the dimensionless peak pressure on the pad increases. This trend indicates that higher supply pressure strengthens the hydrostatic support from the recess and reduces the journal displacement required to balance the applied load. This effect is particularly important for LCH_4_-lubricated bearings because the low viscosity of LCH_4_ can limit purely hydrodynamic pressure generation. The friction coefficient shows only a slight increase with the supply pressure ratio, indicating that enhanced hydrostatic support improves the load-carrying capacity without a substantial friction penalty. When the supply pressure ratio increases from 1.5 to 2, the journal eccentricity ratio decreases from 0.741 to 0.545 (an approximately 26.4% drop) whereas a further increase from 2 to 2.5 results in a smaller reduction from 0.545 to 0.473 (an approximately 13.2% drop). The corresponding dimensionless peak temperature rise shows reductions of approximately 7% and 0.5%, respectively. Overall, increasing the supply pressure ratio from 1.5 to 2 considerably improves the load-carrying capacity and reduces the peak temperature rise, whereas further increases beyond 2 provide only marginal benefits. Therefore, the supply pressure ratio should be selected by balancing bearing performance against supply system pressure requirement and turbopump design complexity.

**Figure 17:**
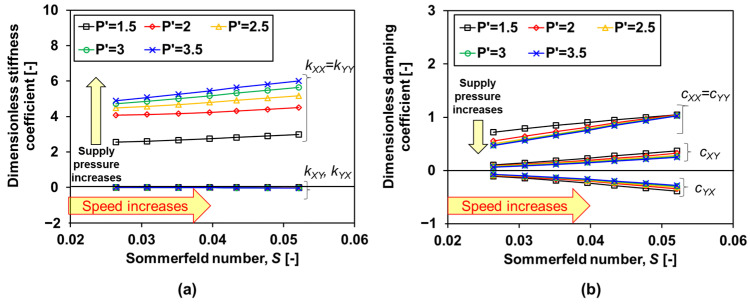
Predicted dimensionless (**a**) stiffness and (**b**) damping coefficients according to the Sommerfeld number under different supply pressure ratio for static load of 5000 N.

With increasing supply pressure ratio, the peak pressure generated in the lubricant film between the shaft and bearing increases, resulting in stiffer fluid-film support. Consequently, the dimensionless direct stiffness increases, while the dimensionless direct damping tends to decrease, as shown in Figure 17. Therefore, the stiffness components show a more pronounced variation than the damping components. Consistent with the static performance trend in Figure 16, the largest change occurs when the supply pressure ratio increases from 1.5 to 2, while further increases produce only limited additional improvement in the dynamic coefficients. Overall, the supply pressure ratio is the dominant design parameter for LCH_4_-lubricated hybrid TPJBs because it most effectively improves load support and direct stiffness with only a minor friction penalty.

### 4.6. Ratio of Recess Area to Total Bearing Area

**Figure 18:**
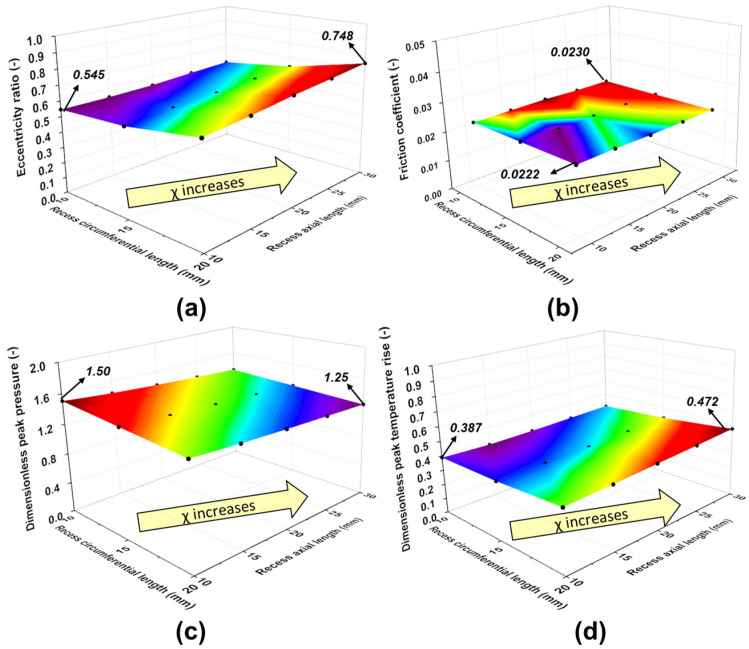
Three-dimensional surface plots of predicted (**a**) journal eccentricity ratio, (**b**) friction coefficient, (**c**) dimensionless peak pressure, and (**d**) dimensionless peak temperature rise at rotating speed of 60,000 rpm and static load of 5000 N at S = 0.052. The contour colors transition from purple to red with increasing values.

**Figure 19:**
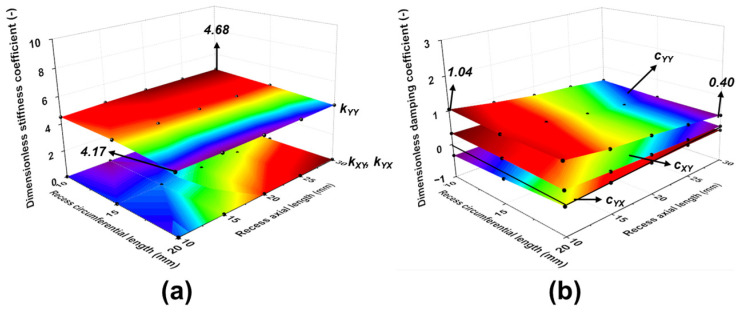
Three-dimensional surface plots of predicted (**a**) dimensionless stiffness and (**b**) damping coefficients at rotating speed of 60,000 rpm and static load of 5000 N at S = 0.052. The contour colors transition from purple to red with increasing values.

The analysis of the ratio of recess area to total bearing area considers the operating conditions of $W_{b}\;=\;5000\;N$, L/D = 1, m = 0.5, ψ = 0.0015, and $P^{\prime }\;=\;2$. Figure 18 and Figure 19 present the corresponding three-dimensional surface plots at a rotational speed of 60,000 rpm and a static load of 5000 N. As shown in Figure 18, increasing the recess area ratio increases the journal eccentricity ratio, decreases the dimensionless peak pressure, and increases the dimensionless peak temperature rise. The journal eccentricity ratio increases from 0.545 to 0.748, corresponding to an increase of approximately 37.2%. The friction coefficient, however, does not exhibit a significant variation with changes in the recess area ratio, and its overall sensitivity remains relatively low compared with the others. The friction coefficient shows a slight decreasing trend when the circumferential recess length increases and the axial recess length decreases. In contrast, the dimensionless peak pressure decreases from 1.50 to 1.25, representing a reduction of approximately 16.7%, while the dimensionless peak temperature rise increases from 0.387 to 0.472, corresponding to an increase of about 22%. This increase occurs because the weakened pressure support raises the journal eccentricity ratio and locally reduces the film thickness in the loaded region, which intensifies shear heating in the LCH_4_ film. A larger recess area reduces the effective land region where the LCH_4_ film develops pressure gradients and expands the recess–film boundary through which the supplied LCH_4_ flows out. This weakens the hydrostatic pressure support and requires a larger journal displacement to support the same load. The increased eccentricity locally reduces the film thickness in the loaded region and intensifies shear heating in the LCH_4_ film, resulting in a higher dimensionless peak temperature rise. Therefore, increasing the recess area ratio does not directly improve the performance of LCH_4_-lubricated hybrid TPJBs; instead, the recess size should be limited to preserve sufficient land area for pressure generation and thermal control.

As shown in Figure 19, the dimensionless stiffness and damping coefficients generally decrease as the recess area ratio increases. For example, the maximum dimensionless direct stiffness reaches 4.68 when the circumferential recess length is the smallest while the axial recess length is the largest. When the circumferential recess length becomes the largest and the axial recess length is reduced, the stiffness decreases to approximately 4.17. The dimensionless stiffness coefficients exhibit greater sensitivity to variations in the circumferential length of the recess than to changes in the axial recess length. Meanwhile, the dimensionless damping coefficients show stronger dependence on variations in the axial recess length than on circumferential changes. As shown in Figure 19b, the maximum dimensionless direct damping coefficient is approximately 1.04 when both the circumferential and axial recess lengths are the smallest. As both recess dimensions increase, the damping coefficient decreases significantly, reaching approximately 0.40 when the circumferential and axial recess lengths are the largest. Furthermore, the cross-coupled damping coefficients remain much smaller than the direct damping coefficients, indicating that the direct damping terms dominate the dissipative response even when the recess geometry changes.

## 5. Conclusions

The present study investigated the performance of a LCH_4_-lubricated hybrid TPJB for reusable turbopumps by extending the hybrid TPJB analytical model with temperature- and pressure-dependent LCH_4_ properties obtained from NIST data. The main conclusions are summarized as follows:

1. The main novelty of this study is the application of LCH_4_ property-based thermohydrodynamic model to a hybrid TPJB. The fourth-order polynomial fitting models for LCH_4_ density and viscosity yielded high fitting accuracy with *R*^2^ = 0.998, allowing for local variations in density and viscosity to be reflected in the pressure and temperature field.
2. The static load and length-to-diameter ratio define the usable load range of the present bearing geometry. Increasing static load raised journal eccentricity, peak pressure, and temperature rise due to intensified pressure buildup and shear heating in the thinner LCH_4_ film. In contrast, increasing *L*/*D* improved the load-carrying capability by distributing the load over a larger pad area, thereby reducing peak pressure and temperature rise. However, the larger shearing area increased frictional loss.
3. The preload and radial clearance ratio require careful design optimization because they directly control the film geometry of the LCH_4_-lubricated bearing. Higher preload and smaller clearance compensate for the limited hydrodynamic pressure generation of low-viscosity LCH_4_ by forming a stronger pressure response and increasing direct dynamic coefficients. However, they also intensify viscous shear and increase peak temperature rise, which can reduce the thermal margin of the cryogenic lubricant film.
4. The supply pressure ratio was the most influential design parameter for the LCH4-lubricated hybrid TPJB. Higher supply pressure directly enhanced hydrostatic support and compensated for the weak hydrodynamic effect of low-viscosity LCH4. As a result, it reduced journal eccentricity, increased direct stiffness, and lowered the peak temperature rise with only a minor increase in friction coefficient. Further increase beyond $P^{\prime }\;=\;2$ produced only marginal improvement. Therefore, the supply pressure ratio should be optimized rather than simply maximized, considering bearing performance, supply-system pressure requirement, and turbopump design complexity.
5. Increasing the recess area ratio weakened the hydrostatic pressure support by reducing the effective land region and expanding the outflow boundary. This effect is critical for LCH_4_ lubrication because the low viscosity of LCH_4_ already limits hydrodynamic pressure generation, making the bearing more dependent on effective hydrostatic support. As the recess area ratio increased, the bearing showed higher eccentricity, higher temperature rise, and lower dynamic coefficients.

The predicted static and dynamic bearing characteristics provide input data for rotor-bearing analysis of reusable LCH_4_ turbopumps. Although the model neglects continuous-film inertia, cavitation, and elastic deformation of the pads, it captures the dominant hydrostatic–hydrodynamic support mechanism and LCH_4_ property effects needed for preliminary design.

## Figures and Tables

**Table 1:** Geometry of hybrid TPJB, pivot, and recess.

Parameter		Value
Hybrid TPJB	Load configuration	LBP
	Journal diameter, *D* (mm)	50
	Pad length, *L* (mm)	50
	Number of pads, *N_pad_*	4
	Pad arc angle, *θ_pad_* (°)	70
	Pad thickness, *t_pad_* (mm)	16
	Radial pad clearance, *C_p_* (μm)	75
	Pivot offset	0.5
	Preload factor, *m*	0.5
Pivot	Pivot type	Rocker-back
	Pivot housing radius, *R_housing_* (mm)	42
	Pivot radius, *R_pivot_* (mm)	36
	Pivot length, *L_pivot_* (mm)	50
	Young’s modulus, *E* (GPa)	200
	Poisson’s ratio, *ν*	0.3
Recess	Recess type	Rectangular
	Recess circumferential length, *l_r_* (mm)	10
	Recess axial length, *b* (mm)	10
	Depth of recess, *D_r_* (mm)	0.2
	Orifice diameter, *d*_o_ (mm)	1
	Orifice discharge coefficient, *C_d_*	0.68

**Table 2:** Lubricant properties, operating conditions and analysis parameters.

System Condition		Value
Lubricant	Lubricant type	LCH_4_
	Ambient pressure, *P_a_* (bar)	200
	Supply pressure, *P_s_* (bar)	400
	Supply temperature, *T_s_* (K)	112
	Ambient temperature, *T_a_* (K)	112
	Density, *ρ*_0_ (kg/m^3^)	438.4
	Viscosity, *μ*_0_ (μPa·s)	150
Bearing parameter ranges and operating conditions	Static load, *W_b_* (N)	500–10,000
	Rotating speed, *N* (rpm)	30,000–60,000
	Length-to-diameter ratio, *L*/*D*	0.5–1.5
	Preload factor, *m*	0.3–0.7
	Radial bearing clearance ratio, *ψ*	0.001–0.002
	Supply pressure ratio, $P^{\prime }\;=\;P_{s}/P_{a}$	1.5–3.5
	Ratio of recess area to total bearing area, *χ*	0.06–0.393
Analysis parameters	Differential step	10^−6^
	Perturbation step ratio	0.001
	Number of circumferential elements	12
	Number of axial elements	20
	Convergence tolerance	10^−6^ (static), 10^−8^ (dynamic)

## Data Availability

The original contributions presented in this study are included in the article. Further inquiries can be directed to the corresponding author.

## Nomenclature

*A*_0_	Orifice area (m^2^)
*b*	Recess axial length (m)
*C_b_*	Radial bearing clearance (m)
*C_d_*	Orifice discharge coefficient (-)
*C_ij_*	Polynomial fitting coefficients
*C_p_*	Radial pad clearance (m)
*C_αβ_*	Damping coefficients (N·s/m), *α*,*β* = *X*,*Y*
*D*	Journal diameter (m)
*D_r_*	Recess depth (m)
*E*	Young’s modulus (Pa)
*F_X_*, *F_Y_*	Resultant of forces in horizontal and vertical direction (N)
*G_X_*, *G_Z_*	Turbulence coefficients in horizontal and vertical direction (-)
*K_αβ_*	Stiffness coefficients (N/m), *α*,*β* = *X*,*Y*
*L*, *L_pivot_*	Bearing and pivot axial length (m)
*M*	Pad moment (N·m)
*N*	Journal rotating speed (rpm)
*N_pads_*	Number of pads (-)
*P*	Pressure (Pa)
*P_dc_*, *P_da_*	Circumferential and axial pressures at the entrances of film land (Pa)
*P**	Dimensionless pressure normalized by ambient pressure (*P*/*P_a_*) (-)
*P* ^′^	Supply pressure ratio normalized by ambient pressure (*P_s_*/*P_a_*) (-)
*Q_l_*	Flux at pad land area (m^2^/s)
*Q_o_*, *Q_r_*	Orifice inflow rate and summation of flow rates at the recess boundaries (m^3^/s)
*R*	Coefficient of determination (-)
*R_b_*, *R_j_*, *R_p_*, *R_housing_*, *R_pivot_*	Radii of bearing, journal, pad curvature, pivot housing, and pivot
*S*	Sommerfeld number (-)
*T*	Temperature (K)
*T_a_*, *T_b_*, *T_j_*, *T_s_*	Ambient, bearing, journal, and supply temperature (K)
*ΔT**	Dimensionless temperature rise relative to reference ambient temperature (-)
*U*	Journal surface speed (m/s)
*V*, *W*	Circumferential and axial direction bulk-flow velocity (m/s)
*W_b_*, *W_X_*, *W_Y_*	Bearing static load, horizontal direction fluid-film force, and vertical direction fluid-film force (N)
*X*, *Y*	Inertial coordinates (m)
*c_p_*	Specific heat capacity at constant pressure (J/kg/K)
*c_αβ_*	Dimensionless damping coefficients (-), *α*,*β* = *X*,*Y*
*d*_0_	Orifice diameter (m)
*e*	Journal eccentricity (m)
*f*	Friction coefficient (-)
*h*, *h_l_*, *h_r_*	Film thickness, pad land film thickness, and recess film thickness (m)
*h_b_*, *h_j_*	Convective heat transfer coefficients to bearing and journal surface (W/m^2^/K)
*k_αβ_*	Dimensionless stiffness coefficients (-), *α*,*β* = *X*,*Y*
*l_r_*	Recess circumferential length (m)
*m*	Preload factor ($1\;-\;C_{b}/C_{p})$ (-)
*m*_0_, *n*_0_	Empirical constants (-)
*p*	Film pressure (Pa)
*p_ratio_*, *p_ratioc_*, *p_ratioa_*	Recess pressure, circumferential, and axial entrance pressure ratios normalized by supply pressure (-)
*r_p_*	Preload (*C_p_*-*C_b_*) (m)
*t*	Time (s)
*t_pad_*	Pad thickness (m)
*x*, *z*	Circumferential and axial coordinates (m)
*x_s_*, *x_e_*	Pad start and end locations in circumferential coordinate (m)
*x_rs_*, *x_re_*, *z_rs_*, *z_re_*	Recess start and end locations in circumferential and axial coordinates (m)
Greek alphabet	
*β_t_*	Thermal expansion coefficient of fluid [K^−1^]
*δ*	Pad tilting angle [rad]
*ε*	Journal eccentricity ratio (*e*/*C_b_*) (-)
*ζ*	Pivot radial displacement (m)
*θ*	Circumferential coordinate (rad)
*θ_c_*, *θ_p_*	Journal attitude angle of journal location and pivot location in circumferential coordinate (rad)
*θ_pad_*	Pad arc angle (rad)
*μ*	Fluid local viscosity (Pa·s)
*μ*_0_	Fluid viscosity at supply temperature (Pa·s)
*ν*	Poisson’s ratio (-)
*ρ*	Fluid local density (kg/m^3^)
*ρ*_0_	Fluid density at supply temperature (kg/m^3^)
*χ*	Ratio of recess area to total bearing area (-)
*ψ*	Radial bearing clearance ratio (*C_b_*/*R_b_*) (-)
*ω*	Journal angular speed (rad/s)
Subscript	
*a*	Ambient condition
*b*	Bearing
*j*	Journal
*l*	Pad land
*o*	Orifice
*r*	Recess
*s*	Supply condition

## Appendix A. Polynomial Fitting Results

Polynomial fitting employed the Curve Fitting Toolbox in MathWorks MATLAB R2025b. The polynomial fitting coefficients (*C_ij_*) represent the influence of temperature and pressure, where the sum of *i* and *j* indicates polynomial order. Equation (1) includes terms up to the fourth order, with each coefficient *i* and *j* ranging from 0 to 4. The polynomial orders higher than four were also examined, but they did not significantly improve the coefficient of determination, while increasing the number of fitting terms and model complexity. Therefore, the fourth-order polynomial form was selected to balance approximation accuracy and model simplicity. Table A1 and Table A2 summarize the resulting fitted coefficients for the proposed model.

**Table A1:** Polynomial fitting coefficients (*C_ij_*) and their units for density fitting model according to temperature and pressure.

**Fitting Coefficient**	*C*_00_	*C*_10_	*C*_01_	*C*_20_	*C*_11_
**Unit**	kg/m^3^	kg/m^3^_·_K	kg/m^3^_·_bar	kg/m^3^_·_K^2^	kg/m^3^_·_K_·_bar
**Value**	497.8	0.9247	0.06999	−0.02626	−0.00036
**Fitting coefficient**	*C*_02_	*C*_30_	*C*_21_	*C*_12_	*C*_03_
**Unit**	kg/m^3^·bar^2^	kg/m^3^·K^3^	kg/m^3^·K^2^·bar	kg/m^3^·K·bar^2^	kg/m^3^·bar^3^
**Value**	−1.5 × 10^−5^	0.000151	−5.5 × 10^−6^	1.84 × 10^−6^	−8.93 × 10^−8^
**Fitting coefficient**	*C*_40_	*C*_31_	*C*_22_	*C*_13_	*C*_04_
**Unit**	kg/m^3^·K^4^	kg/m^3^·K^3^_·_bar	kg/m^3^·K^2^·bar^2^	kg/m^3^·K·bar^3^	kg/m^3^·bar^4^
**Value**	−3.9 × 10^−7^	1.13 × 10^−7^	−3.4 × 10^−8^	4.18 × 10^−9^	−2.1 × 10^−10^

**Table A2:** Polynomial fitting coefficients (*C_ij_*) and their units for viscosity fitting model according to temperature and pressure.

**Fitting Coefficient**	*C*_00_	*C*_10_	*C*_01_	*C*_20_	*C*_11_
**Unit**	μPa·s	μPa·s/K	μPa·s/bar	μPa·s/K^2^	μPa·s/K·bar
**Value**	3768	−98.63	3.825	1.007	−0.0733
**Fitting coefficient**	*C*_02_	*C*_30_	*C*_21_	*C*_12_	*C*_03_
**Unit**	μPa·s/bar^2^	μPa·s/K^3^	μPa·s/K^2^·bar	μPa·s/K·bar^2^	μPa·s/bar^3^
**Value**	0.00022	−0.00462	0.000479	−3.86 × 10^−6^	7.83 × 10^−8^
**Fitting coefficient**	*C*_40_	*C*_31_	*C*_22_	*C*_13_	*C*_04_
**Unit**	μPa·s/K^4^	μPa·s/K^3^·bar	μPa·s/K^2^·bar^2^	μPa·s/K·bar^3^	μPa·s/bar^4^
**Value**	7.92 × 10^−6^	−1.03 × 10^−6^	9.69 × 10^−9^	2.9 × 10^−10^	−4.2 × 10^−11^

## References

[1] Okayasu A., Ohta T., Kamijyo A., Yamada H. (2002). Key Technology for Reusable Rocket Engine Turbopump. Acta Astronaut..

[2] Xu J., Li C., Miao X., Zhang C., Yuan X. (2020). An Overview of Bearing Candidates for Next Generation Reusable Liquid Rocket Turbopumps. Chin. J. Mech. Eng..

[3] Hannum N., Nielson C. Performance and Application of High-Speed Long-Life LH2 Hybrid Bearings for Reusable Rocket Turbomachinery. Proceedings of the 19th Joint Propulsion Conference.

[4] San Andrés L. (1995). Thermohydrodynamic Analysis of Fluid Film Bearings for Cryogenic Applications. J. Propuls. Power.

[5] Yang Z., San Andrés L., Childs D.W. (1996). Thermal Effects in Liquid Oxygen Hydrostatic Journal Bearings. Tribol. Trans..

[6] Yang Z., San Andrés L., Childs D.W. (1995). Thermohydrodynamic Analysis of Process-Liquid Hydrostatic Journal Bearings in Turbulent Regime, Part II: Numerical Solution and Results. J. Appl. Mech..

[7] Franchek N.M., Childs D.W., San Andrés L. (1995). Theoretical and Experimental Comparisons for Rotordynamic Coefficients of a High-Speed High-Pressure Orifice-Compensated Hybrid Bearing. J. Tribol..

[8] Heshmat H. A Feasibility Study on Use of Foil Bearings in Cryogenic Turbopumps. Proceedings of the 27th Joint Propulsion Conference.

[9] Behera S.K., Bohra D.G., Khamari D.S., Kumar J., Bassan G.D. Feasibility Study of Foil Journal Bearing for LH2 Turbopump Used in Cryogenic Engine. Proceedings of the 20th ISME Conference on Advances in Mechanical Engineering.

[10] San Andrés L. (1995). Turbulent Flow Foil Bearings for Cryogenic Applications. J. Tribol..

[11] Behera S.K., Khamari D.S. Numerical Investigation on Stiffness and Damping of Gas Foil Journal Bearings Supporting High-Speed Rotor of Cryogenic LH2 Turbopumps. Proceedings of the 11th International Conference on Industrial Tribology.

[12] San Andrés L. (1996). Turbulent Flow Flexure-Pivot Hybrid Bearings for Cryogenic Applications. J. Tribol..

[13] Latimier C. (2024). Numerical Analysis of Raptor Engine Combustion Chamber. Ph.D. Thesis.

[14] Mehdi S.M., Jeong S.Y., Kim T.H. (2022). Influence of Recess Dimensions and Jacking Oil Flow Rate on Performance of Tilting Pad Journal Bearings with Jacking Oil Mechanism. Tribol. Trans..

[15] Mehdi S.M., Kim T.H. (2022). Computational Model Development for Hybrid Tilting Pad Journal Bearings Lubricated with Supercritical Carbon Dioxide. Appl. Sci..

[16] Hagemann T., Pfeiffer P., Schwarze H. (2018). Measured and Predicted Operating Characteristics of a Tilting-Pad Journal Bearing with Jacking-Oil Device at Hydrostatic Hybrid and Hydrodynamic Operation. Lubricants.

[17] Chaomleffel J.P., Nicolas D. (1986). Experimental Investigation of Hybrid Journal Bearings. Tribol. Int..

[18] Piunti M., Shytani A., Persico F., Pasetti S. (2022). Preliminary Design of a Raptor-Like Engine.

[19] Lemmon E.W., McLinden M.O., Huber M.L. NIST Reference Fluid Thermodynamic and Transport Properties—REFPROP. https://webbook.nist.gov/chemistry/fluid/.

[20] Balbahadur A.C. (2001). A Thermoelastohydrodynamic Model of the Morton Effect Operating in Overhung Rotors Supported by Plain or Tilting Pad Journal Bearings. Ph.D. Thesis.

[21] Young W.C., Budynas R.G. (2002). Roark’s Formulas for Stress and Strain.

[22] Kirk R.G., Reedy S.W. (1988). Evaluation of Pivot Stiffness for Typical Tilting-Pad Journal Bearing Designs. J. Vib. Acoust..

[23] Nicholas J.C., Wygant K.D. Tilting Pad Journal Bearing Pivot Design for High Load Applications. Proceedings of the 24th Turbomachinery Symposium.

[24] Mehdi S.M., Jang K.E., Kim T.H. (2018). Effects of Pivot Design on Performance of Tilting Pad Journal Bearings. Tribol. Int..

[25] Hashimoto H., Wada S., Yamamoto S. (1985). An Influence of Fluid Inertia Forces on the Dynarnic Characteristics of Tilting-pad Journal Bearings in Turbulent Flow. Bull. JSME.

[26] SanAndrés L. Notes 8: Turbulence in Thin Film Flows. https://rotorlab.tamu.edu/me626/Notes_pdf/Notes08%20Turbulence%20Flow%20in%20Thin%20Films.pdf.

[27] Hirs G.G. (1973). A Bulk-Flow Theory for Turbulence in Lubricant Films. J. Lubr. Technol..

[28] SanAndrés L. Notes 10: Thermohydrodynamic Bulk-Flow Model in Thin Film Lubrication. https://rotorlab.tamu.edu/me626/Notes_pdf/Notes10%20THD_BulkFlowModel_09.pdf.

[29] Bou-Said B., Chaomleffel J.P. (1989). Hybrid Journal Bearings: Theoretical and Experimental Results. J. Tribol..

[30] San Andrés L. Notes 7: Thermal Analysis of Finite Length Journal Bearings Including Fluid Inertia. https://rotorlab.tamu.edu/me626/Notes_pdf/Notes07%20Thermal%20Anaysis%20JBs%20w%20examples.pdf.

